# SETD1A Promotes Proliferation of Castration-Resistant Prostate Cancer Cells via FOXM1 Transcription

**DOI:** 10.3390/cancers12071736

**Published:** 2020-06-30

**Authors:** Liu Yang, Mingli Jin, Sung Jean Park, Seung-Yong Seo, Kwang Won Jeong

**Affiliations:** Gachon Research Institute of Pharmaceutical Sciences, College of Pharmacy, Gachon University, 191 Hambakmoero, Yeonsu-gu, Incheon 21936, Korea; yangliu0909@gc.gachon.ac.kr (L.Y.); mingli1217@hanmail.net (M.J.); psjnmr@gachon.ac.kr (S.J.P.); syseo@gachon.ac.kr (S.-Y.S.)

**Keywords:** SETD1A, FOXM1, prostate cancer, castration-resistant

## Abstract

Androgen deprivation therapy eventually leads to the development of castration-resistant prostate cancer (CRPC). Here, we demonstrate for the first time that the histone H3K4 methyltransferase SETD1A is a major regulator for the proliferation of metastatic CRPC (mCRPC). The expression of SETD1A was significantly correlated with the survival rate of patients with prostate cancer. SETD1A, which is expressed at a higher level in mCRPC than in primary prostate cancer cells, promotes the expression of *FOXM1*, a gene encoding a cell proliferation-specific transcription factor. SETD1A is recruited to the promoter region of *FOXM1* (forkhead box M1) upon binding to E2F1, a protein that regulates the transcription of FOXM1 and contributes to the trimethylation of H3K4 in the *FOXM1* promoter region. In addition, SETD1A is essential for the expression of stem cell factor (e.g., *OCT4,* octamer-binding transcription factor 4) and stem cell formation in mCRPC, suggesting the importance of SETD1A expression in mCRPC tumor formation. Notably, poor prognosis is associated with high expression of the SETD1A–FOXM1 pair in clinical data sets. Therefore, our study suggests that SETD1A plays an important role in the proliferation of mCRPC by regulating *FOXM1* transcription.

## 1. Introduction

As the androgen receptor (AR) pathway is critical for the progression and development of prostate cancer, androgen deprivation, which leads to the blockage of AR activity, is an effective therapy for the initial treatment of prostate cancer. However, patients invariably relapse and eventually develop castration resistance [1,2]. Metastatic castration-resistant prostate cancer (mCRPC) is related to a worse prognosis and is known to be clinically incurable [3,4]. Therefore, deciphering the underlying mechanism for castration resistance is critical and may provide a foundation for developing future therapeutic options for mCRPC.

Many previous studies have attempted to find target molecules for CRPC therapy. GATA2 (GATA-binding protein 2) and FOXA1 (forkhead box A1) have been proposed as important regulators of AR splice variant-driven transactivation that has been shown to be associated with CRPC [5]. GATA2 induces tumorigenicity and resistance to chemotherapy by giving rise to the GATA2–IGF2 (insulin like growth factor 2) axis in CRPC [6]. FOXA1 mediates the inhibition of TGF-β (transforming growth factor beta) signal transduction [7] and promotes the chromatin binding of AR in the absence of androgen [8]; these play a central role in the development of CRPC. ONECUT2 (OC2) has also been reported as a master regulator in metastatic CRPC. Additionally, with the help of computational modeling, several transcription factors (e.g., EZH2, FOXM1, PAX5, E2F7, POU5F1) have been predicted to be significantly more active in mCRPC that in primary tumors [9].

Epigenetic changes, including histone modification and DNA methylation, play a pivotal role in carcinogenesis and tumor progression. These epigenetic alterations are controlled and coordinated by diverse epigenetic regulators [10]. In recent years, targeting epigenetic regulatory factors has been considered as another approach for identifying the key molecules promoting the proliferation of castration-resistant prostate cancer. It has been reported that LSD1 (lysine-specific demethylase 1A) induces the progression of prostate cancer by mediating epigenetic reprogramming [11] or by activating a lethal prostate cancer gene network [12]. In addition, the polycomb group (PcG) protein, BMI1, is known to play an important role in the development of mCRPC by regulating AR independently of the PRC1 complex [13]. Concurrently, EZH2 (enhancer of zeste homolog 2), another PcG protein, has been indicated to play a role in mCRPC progression. Upon phosphorylation, EZH2 switches from a PRC2-repressor to a transcriptional coactivator of AR, which is required for the progression of mCRPC. Additionally, the methyltransferase domain of EZH2 is also required for its oncogenic function in mCRPC [14]. 

SETD1A is a histone H3K4 methyltransferase that is a member of the human SET domain family [15]. Best characterized as a transcriptional coactivator, SETD1A interacts with a conserved core module of four proteins (namely WRD5, RBBP5, ASH2L, and DPY30) to methylate histone H3K4 [16,17]. Aberrant expression of SETD1A results in the pathogenesis of human diseases, including acute myeloid leukemia (AML) [18], colorectal cancer [19], and triple-negative breast cancer [20]. Recently, our study demonstrated that SETD1A regulates the recruitment of estrogen receptor (ER)α and the formation of an accessible chromatin structure in the enhancer region of ERα target genes to promote cell survival in ERα positive breast cancer. Most importantly, we reported that SETD1A is involved in the survival of tamoxifen-resistant cells through the regulation of non-ER target gene expression [21]. In prostate cancer cells, SETD1A is reported to regulate cell cycle and tumor progression [20]. However, the functional contribution of SETD1A and its underlying mechanism in prostate cancer and mCRPC remains unclear. 

Here, we investigated the effect of SETD1A expression on genes whose expression changes drastically during the development of mCRPC using prostate cancer cells. We determined the expression of SETD1A in prostate cancer tissues and its relation with relapse-free survival (RFS). We then identified that FOXM1 is the molecular target responsible for the modulation of SETD1A function with respect to cell proliferation and migration in mCRPC. We have also described the mechanism by which SETD1A regulates FOXM1 expression. Taken together, our data provide a conceivable rationale for targeting the SETD1A–FOXM1 axis in patients with prostate cancer, including mCRPC.

## 2. Results

### 2.1. Overexpression of SETD1A Correlates with Poor Prognosis in Prostate Tumors

**Figure 1:**
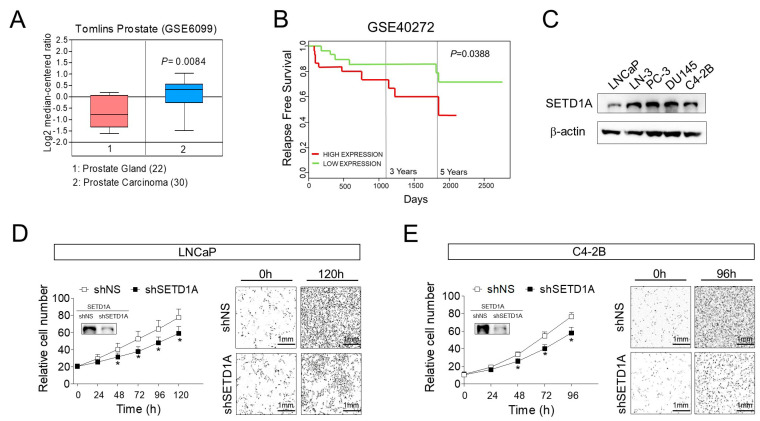
Overexpression of SETD1A in prostate cancer and its effect on cell growth. (**A**) The expression of *SETD1A* mRNA (messenger RNA) was compared between normal prostate tissue (pink box) and prostate carcinoma (blue box) using public dataset (GSE6099). (**B**) Kaplan–Meier relapse-free survival plot of patients with prostate cancer created using the PROGgeneV2 platform. Patients were stratified based on median into SETD1A-high and SETD1A-low subgroups and analyzed as indicated. (**C**) Protein level of SETD1A in multiple prostate cancer cell lines. (**D**,**E**) Cell proliferation in shRNA (short hairpin RNA) -silenced LNCaP (**D**) and C4-2B cells (**E**) grown in complete culture medium was analyzed using a live cell imaging system in 6-well plates. Each value represents mean ± S.D (standard deviation). * *p* < 0.05 vs. shNS (non-specific shRNA) control. The panels on the right side of each proliferation graph show representative images of corresponding cell lines in both conditions at indicated time points that were randomly selected from the 16 sites (as described in Materials and Methods).

We examined the expression of SETD1A in prostate cancer tissue. The expression of *SETD1A* mRNA (GSE6099) was found to be higher in prostate tumors than that in normal prostate tissue (Figure 1A). In addition, patients with high SETD1A expression showed lower RFS (relapse free survival) compared to patients with low SETD1A expression (Figure 1B). These findings suggested the clinical relevance of SETD1A in prostate cancer and led us to assume that SETD1A may play a pivotal role in the progression of prostate cancer. Consistent with this hypothesis, we observed that growth of AR-dependent prostate cancer cells (LNCaP), as well as AR-independent prostate cancer cells (C4-2B, PC-3, DU145, and LNCaP-LN3), was significantly inhibited upon depletion of SETD1A in these cell lines using siRNA or shRNA (Figure 1C–E and Figure S1). These results suggest that SETD1A plays an important role in the proliferation of prostate cancer.

### 2.2. Regulation of FOXM1 Target Genes by SETD1A in mCRPC

**Figure 2:**
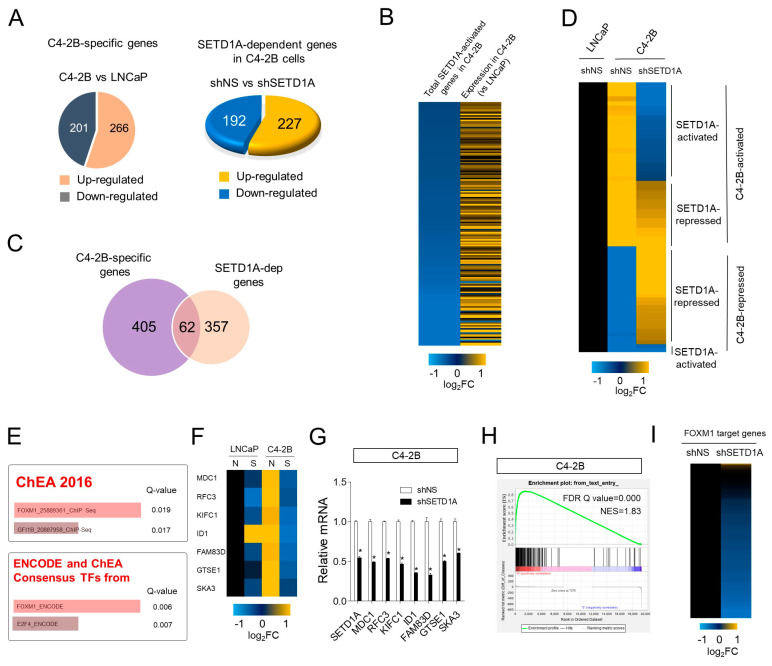
Regulation of FOXM1 target genes by SETD1A in metastatic castration-resistant prostate cancer (mCRPC). (**A**) Pie graphs showing numbers of differentially expressed genes in LNCaP and C4-2B cells (left) and genes whose expression was dramatically changed in response to SETD1A knockdown in C4-2B cells (right). (**B**) Heat map showing that most of the total SETD1A-activated genes were overexpressed in C4-2B cells compared to that in LNCaP cells. (**C**) Venn diagram showing overlap of C4-2B specific genes and SETD1A dependent genes in C4-2B cells. (**D**) Heat map for the genes that were overlapping in the Venn diagram in Figure 2C showing the expression pattern of SETD1A-dependent genes among C4-2B specific genes. (**E**) Gene ontology analysis using SETD1A-activated genes among the C4-2B-activated genes. The length of the bars represents the combined score from the Fisher exact test. Q-values suggest the statistical significance for specific terms. (**F**) Heat map of FOXM1-target gene expression obtained from Enrichr analysis. N, shNS; S, shSETD1A (**G**) Validation of RNA-seq results by RT-qPCR (reverse transcript-quantitate polymerase chain reaction) analysis showing the mRNA level of FOXM1 target genes in C4-2B cells treated with shNS vs. those treated with shSETD1A. The mRNA levels were normalized to that of *18S* rRNA. Each value represents mean S.D. * *p* < 0.05. (**H**) GSEA (gene set enrichment analysis) analysis using the FOXM1-target gene set. Upward deflection suggests enrichment of the FOXM1 signaling signature in SETD1A activated genes (FDR, false discovery rate = 0.000). (**I**) The effect of SETD1A depletion on the expression of FOXM1 target gene set in C4-2B cells.

To identify SETD1A-target genes involved in the survival of mCRPC, we observed the changes in mRNA expression patterns after depletion of SETD1A in LNCaP and C4-2B cell lines. Compared to the LNCaP cells, 467 genes were differently expressed in the C4-2B cells, including 266 upregulated genes and 201 downregulated genes (Figure 2A, left panel). In addition, 419 genes (227 upregulated and 192 downregulated) regulated by SETD1A in C4-2B cells were also identified (Figure 2A, right panel). Most of these SETD1A-activated genes were overexpressed in C4-2B cells compared to that in LNCaP cells (Figure 2B). From the above two results, we identified 62 genes among C4-2B cell-specific genes that were differentially expressed by SETD1A depletion (Figure 2C,D). As SETD1A is known to be a transcriptional coactivator, the genes activated by SETD1A were selected from the genes differentially expressed in C4-2B cells for further analysis. Pathway analysis revealed that SETD1A-dependent genes were enriched in the cell cycle pathway (Q = 0.0000 KEGG) (Figure S2). From these results, we could assume that SETD1A may play an important role in the proliferation of castration-resistant cancer cells.

Next, we sought to identify upstream transcription factors that regulate the expression of C4-2B specific genes regulated by SETD1A. In in silico analysis, it was predicted that FOXM1 could function as a potential transcriptional regulator (Figure 2E,F). The effect of SETD1A-depletion on representative FOXM1-target genes was confirmed by RT-qPCR (reverse transcript-quantitate polymerase chain reaction) in C4-2B cells (Figure 2G). In addition, GSEA (gene set enrichment analysis) using FOXM1 regulatory gene set derived from previous reports [22,23] revealed that genes downregulated due to SETD1A knockdown in C4-2B cells were dramatically enriched in a set of FOXM1-target genes (Figure 2H), and the expression of most of these genes was inhibited by SETD1A-depletion (Figure 2I). The effect of SETD1A on the expression of FOXM1 target genes was similarly observed in LNCaP cell lines. However, the level of SETD1A effect was more extensive in C4-2B cells than that in LNCaP cells (Figure 1D), whereby more FOXM1 target genes were more markedly inhibited by SETD1A-depletion in C4-2B cells (Figure S3). 

It is well documented that AR and steroid hormones are required for prostate cancer cell growth. Our RNA-seq results showed no significant effect of SETD1A on AR target gene expression (Figure S2). Although persistent AR signaling is still considered the main cause of mCRPC [3], the AR-independent pathway was also proved critical for mCRPC. We further investigated the effect of SETD1A on AR signaling in LNCaP and C4-2B cells (Figures S4 and S5). In both of these cell lines, the overlap between the AR-mediated genes, selected from a previous report [24], and SETD1A-dependent genes was extremely limited. These results were reconfirmed by RT-qPCR performed for representative AR target genes. These results suggest that SETD1A is independent of AR signaling for the proliferation of mCRPC.

### 2.3. Overexpressed SETD1A in C4-2B Regulates FOXM1 Expression

**Figure 3:**
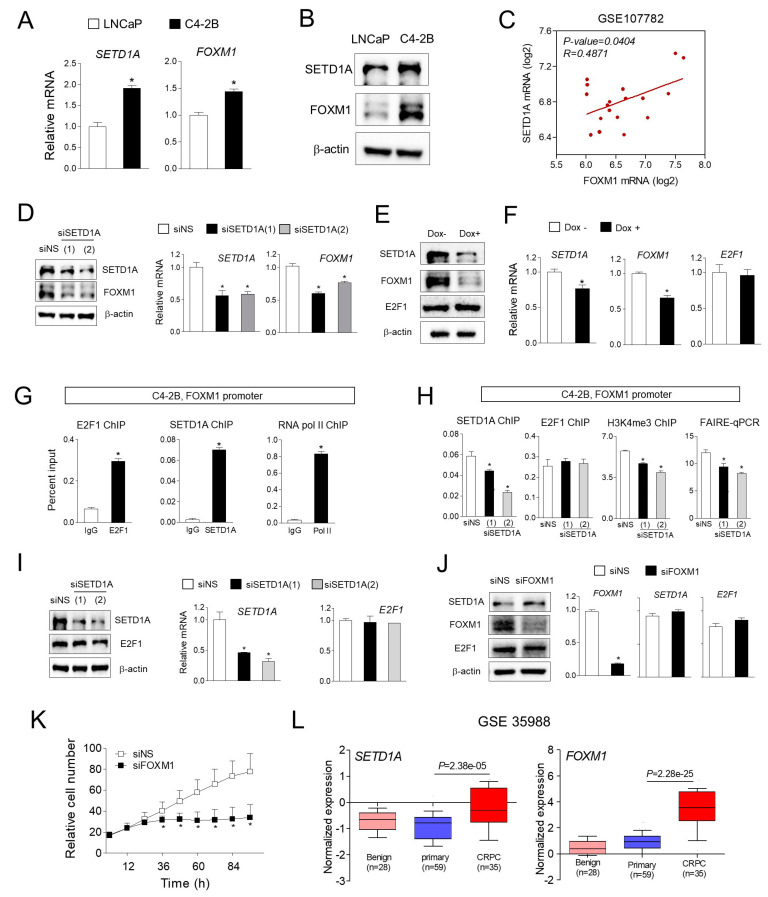
SETD1A regulates the transcription of *FOXM1* gene. (**A**) The results of RT-qPCR performed using total RNA isolated from LNCaP and C4-2B cells showing the relative mRNA levels of *SETD1A* and *FOXM1* in C4-2B cells compared with those in LNCaP cells. The mRNA levels were normalized to that of 18S rRNA. Each value represents mean ± S.D. * *p* < 0.05. (**B**) Protein levels of SETD1A and FOXM1 in whole-cell lysate of LNCaP and C4-2B cells. (**C**) Pearson correlation analysis of SETD1A and FOXM1 expression in LNCaP and C4-2B. Plotted data showing the level of log_2_ mRNA expression in GSE107782 dataset. (**D**) C4-2B cells were transfected with siNS (non-specific siRNA) or siRNA targeting SETD1A (siSETD1A)(1) and siSETD1A(2). After 72 h of transfection, total RNA was extracted by Trizol and analyzed by RT-qPCR, and the mRNA levels were normalized to that of 18S rRNA. Each value represents mean ± S.D. * *p* < 0.05. And total cell lysate was used to compare the protein level by Western blot. (**E**,**F**) Dox-induced SETD1A knockdown affected the protein and mRNA levels of FOXM1. (**G**) ChIP (chromatin immunoprecipitation) showing the recruitment of SETD1A and RNA Pol II to the FOXM1 promoter region. Enrichment of IgG, SETD1A, E2F1, or RNA Pol II was calculated as a percentage of input. Each value represents mean ± S.D. * *p* < 0.05. (**H**) Enrichment of SETD1A, E2F1, or H3K4me3 at FOXM1 promoter region determined by ChIP in siNS (non-specific siRNA) or siSETD1A transfected C4-2B cells. Formaldehyde-Assisted Isolation of Regulatory Elements (FAIRE)-qPCR (quantitate polymerase chain reaction) analysis was performed for the promoter region of FOXM1 in control or SETD1A-depleted C4-2B cells. Data was calculated as a percentage of input. Each value represents mean ± S.D. * *p* < 0.05. (**I**) C4-2B cells transfected with siNS or siSETD1A. Cells were lysed, and Western blotting was performed using indicated antibodies, and mRNA level was analyzed by RT-qPCR. The mRNA levels were normalized to that of 18S rRNA. Each value represents mean ± S.D. * *p* < 0.05. (**J**,**K**) The effect of FOXM1 depletion on the proliferation of C4-2B cells. Cells were transfected with siRNA targeting FOXM1 or control siNS, and the indicated gene expression levels were measured by Western blotting and RT-qPCR. The mRNA levels were normalized to that of 18S rRNA. Each value represents mean ± S.D. * *p* < 0.05. Cell growth was determined using IncuCyte. * *p* < 0.05. (**L**) The normalized expression of SETD1A and FOXM1 in benign, primary, and CRPC tissue samples (GSE 35988).

To explore the mechanism of activation of FOXM1 target gene expression by SETD1A in C4-2B cells, we first compared the expression levels of SETD1A and FOXM1 in LNCaP and C4-2B cells. Both mRNA and protein levels of SETD1A and FOXM1 were upregulated in C4-2B cells compared to those in LNCaP cells (Figure 3A,B). The correlation between SETD1A and FOXM1 expression was verified in the human the prostate cancer gene expression data set. In test sets containing data of LNCaP and C4-2B (GSE107782) [25], and other types of prostate cancer cell lines (GSE50936), we found that *FOXM1* mRNA expression was significantly and positively correlated with that of SETD1A (Figure 3C, and Figure S6). Next, *FOXM1* gene expression was reduced upon SETD1A depletion using two independent siRNAs targeting different SETD1A coding regions (Figure 3D). Similar results were obtained by using inducible shRNA targeting different sites in the *SETD1A* mRNA (Figure 3E,F). Previous studies on MCF-7 cells have reported that E2F1 regulates *FOXM1* transcription by binding to the promoter region of the *FOXM1* gene [26]. In our study, both E2F1 and SETD1A were recruited to the *FOXM1* promoter region (Figure 3G). Given that SETD1A is known to be a histone H3K4 methyltransferase, knockdown of SETD1A significantly reduced the levels of histone H3K4me3 and chromatin accessibility at the *FOXM1* promoter site (Figure 3H) without affecting the levels of E2F1 (Figure 3F,H,I). In contrast, knockdown of FOXM1 resulted in significant inhibition of the proliferation of C4-2B cells without affecting the expression level of SETD1A and E2F1 (Figure 3J,K). As expected, we observed that knockdown of E2F1 also led to the inhibition of FOXM1 expression at the mRNA and protein levels, reduced SETD1A recruitment to *FOXM1* promoter, and suppressed the growth of C4-2B cells (Figure S7). Notably, the depletion of E2F1 (Figure S7A,B) resulted in decreased recruitment of SETD1A to the *FOXM1* promoter (Figure S7C), suggesting the possible role of E2F1 in the recruitment of SETD1A.

FOXM1 is known as a master regulator of cell proliferation, self-renewal, and tumorigenesis in various cancer cells [27,28]. We observed that the expression of both *SETD1A* and *FOXM1* was increased in CRPC samples compared to that in primary prostate cancer in two independent clinical data sets (Figure 3L and Figure S8). Together with the positive correlation between the expression of SETD1A and FOXM1 (Figure 3C), these results suggest that SETD1A is upregulated in castration-resistant C4-2B cells compared to that in primary LNCaP cells and regulates *FOXM1* gene expression at the transcription level upon recruitment to the *FOXM1* promoter region. 

### 2.4. SETD1A Interacts with E2F1 in C4-2B Cells

**Figure 4:**
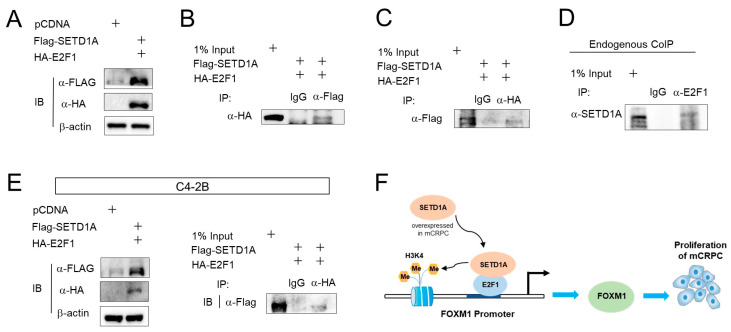
SETD1A interacts with E2F1 in C4-2B and LNCaP cells. (**A–C**) HA(hemagglutinin)-tagged E2F1 and flag-tagged SETD1A were co-expressed in LNCaP cells, and their protein levels were measured 72 h later by Western blotting (**A**) HA-E2F1 and flag-SETD1A were co-expressed in LNCaP cells. Immunoprecipitation was performed using an anti-flag antibody (**B**) or an anti-HA antibody (**C**) Immunoblotting was performed using indicated antibodies. (**D**) Endogenous coimmunoprecipitation (CoIP) of E2F1 and SETD1A in LNCaP cells. (**E**) CoIP of HA-E2F1 and flag-SETD1A in C4-2B cells. Western blot results showing the higher expression of the two proteins after overexpression (left panel). Co-immunoprecipitated SETD1A was detected using anti-flag antibody (right panel). (**F**) The mechanism of crosstalk between SETD1A, FOXM1, and E2F1, and the effect on prostate cancer cells.

Previously, we showed that E2F1 is required for the recruitment of SETD1A to the *FOXM1* gene (Figure S7C). In an attempt to understand the mechanism by which SETD1A is recruited to the *FOXM1* promoter, we conducted coimmunoprecipitation (CoIP) experiments. The expression vectors for flag-tagged SETD1A and HA (hemagglutinin)-tagged E2F1 were transfected into LNCaP cells (Figure 4A). Anti-flag antibody was used to pull-down SETD1A in LNCaP cells, and immunoblot analysis showed that E2F1 was co-precipitated with SETD1A (Figure 4B). Another CoIP experiment performed to analyze the above in the reverse direction (pull-down of E2F1 and immunoblotting for SETD1A) showed a similar result (Figure 4C). Thus, when endogenous E2F1 was pulled down using the E2F1 antibody, SETD1A was co-precipitated with E2F1 in LNCaP cells (Figure 4D). The same interaction between SETD1A and E2F1 was observed in C4-2B cells (Figure 4E). These data suggest that SETD1A is associated with E2F1 in prostate cancer cells, and it is recruited to the *FOXM1* promoter upon binding to E2F1 (Figure 4F). 

### 2.5. SETD1A Promotes Proliferation, Migration, and Invasion of C4-2B Cells

**Figure 5:**
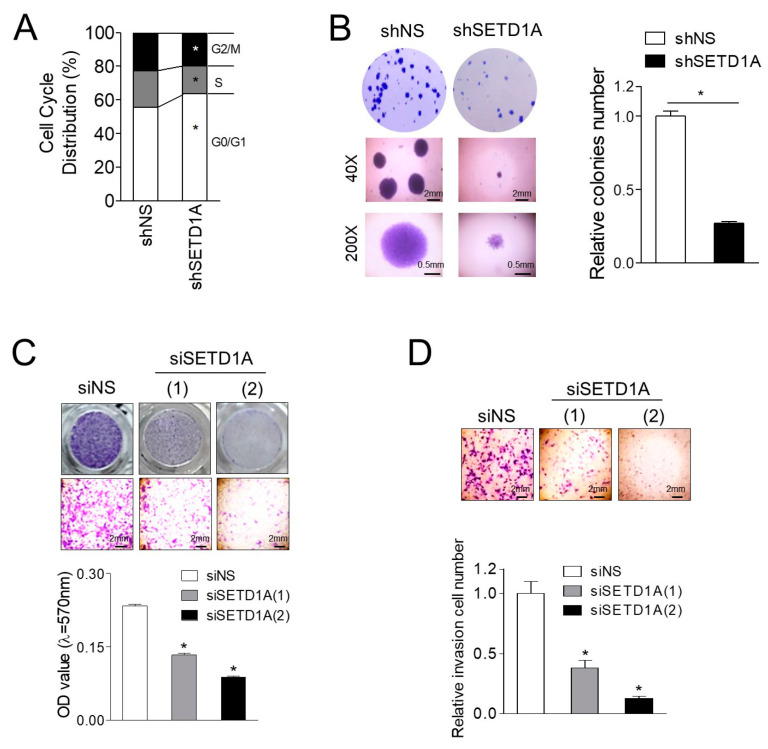
SETD1A promotes proliferation, migration, and invasion of C4-2B cells. (**A**) C4-2B cells were transfected with shNS or shSETD1A, and the cell cycle was analyzed by propidium iodide (PI) staining and flow cytometry. The proportion of cells in different phases of the cell cycle is shown (*n* = 3). (**B**) shNS or shSETD1A-transfected C4-2B cells were analyzed for their ability to proliferate in a growth medium containing 0.35% agar with the formation of multicellular colonies imaged at ×40 (middle panels) or ×200 (bottom panels) magnification after 14 days of incubation in 6-well plates in triplicate. (**C**) Migration assay was performed using cells transfected with siNS or siSETD1A to determine the migration ability of C4-2B cells using transwell chambers. The bar graph represents the quantitative analysis of the stained, migrated cells analyzed using a microplate reader at 570 nm. (**D**) Matrigel invasion assays in transwell using C4-2B cells transfected with siNS or siSETD1A. Data are represented as mean ± S.D; * *p* < 0.05.

FOXM1 is known to be involved in drug resistance in mCRPC [29]. Given that FOXM1 is known as a key molecule that controls the cell cycle process [30], and our pathway analysis revealed that SETD1A-dependent genes were enriched in the cell cycle pathway (Figure S2), we investigated the effect of SETD1A on the cell cycle in C4-2B and LNCaP cells. Compared to the control cells, a significant increase in G0/G1 phase and a decrease in S and G2/M phases were observed in SETD1A-depleted cells (Figure 5A and Figure S9A,B). As SETD1A affects the growth of prostate cancer cells (Figure 1E,F and Figure S1), we investigated whether SETD1A influences colony formation. LNCaP and C4-2B cells with depletion of SETD1A formed colonies in soft agar that were smaller in number and size compared to those of control cells (Figure 5B and Figure S9C). In addition, we tested the role of SETD1A in cell migration and invasion. The transwell migration assay was performed to determine the migratory capacity of SETD1A-depleted cells. The migratory capacities of LNCaP cells and C4-2B cells were significantly reduced due to the depletion of SETD1A (Figure 5C, Figure S9D,E). Subsequently, the effect of SETD1A on the invasion of C4-2B cells was also determined by matrix invasion assay. We observed a drastic reduction in cell invasion in cells depleted for SETD1A using siRNA or shRNA (Figure 5D and Figure S10). These data suggest that SETD1A plays an important role in regulating the proliferation, migration, and invasion of prostate cancer cells, indicating its potential role in the treatment of prostate cancer. 

### 2.6. The SETD1A–FOXM1 Axis Is Associated with Poor Prognosis of Prostate Cancer

**Figure 6:**
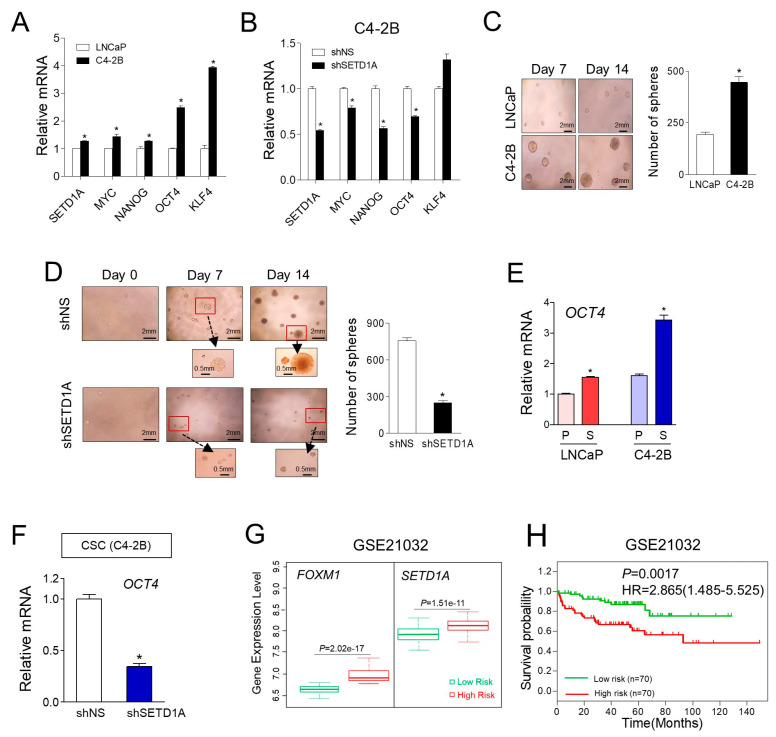
The SETD1A–FOXM1 axis is associated with poor prognosis of prostate cancer. (**A**) The expression of cancer stem cell markers (e.g., *MYC*, *NANOG*, *OCT4,* and *KLF4*) in LNCaP and C4-2B cells was determined by RT-qPCR. (**B**) The expression of cancer stem cell markers was analyzed by RT-qPCR in C4-2B cells transfected with shNS or shSETD1A. (**C**) Formation of cancer stem cell spheres by LNCaP and C4-2B cells. (**D**) The effect of SETD1A on sphere formation in C4-2B cells. The total number of spheres was counted under a microscope (*n* = 3). Data are represented as mean ± S.D. * *p* < 0.05. (**E**) Comparison of *OCT4* expression in parental (P) vs. CSC sphere (S) of LNCaP and C4-2B cells. (**F**) The expression of *OCT4* in cancer stem cell (CSC) spheres derived from C4-2B cells transfected with shNS or shSETD1A. The mRNA levels of the *OCT4* were analyzed by RT-qPCR. Data are represented as mean ± S.D. * *p* < 0.05. (**G**) Comparison of expression of SETD1A and FOXM1 in the low-risk (green box) and high-risk (red box) groups of patients with prostate cancer (GSE21032). (**H**) Kaplan–Meier recurrence-free survival curves represent the prognostic ability of SETD1A and FOXM1 signatures. Low- and high-risks groups are shown in green and red, respectively.

mCRPC cells exhibit several characteristics that are similar to cancer stem cells [31]. In prostate cancer, FOXM1 has been reported to regulate cancer stem cells (CSCs) through regulating UHRF1 gene expression and related taxane resistance [32]. Inhibition of FOXM1 effectively reduced cancer stemness characteristic in enzalutamide-resistant CRPC [33]. Our results also showed that the expression of cancer stem cell marker genes (e.g., *MYC, NANOG, OCT4*, and *KLF4*) was higher in C4-2B cells than that in LNCaP cells and it was markedly decreased by the silencing of SETD1A (Figure 6A,B). We investigated whether SETD1A regulated the formation of spheres of mCRPC. First, we found that the tumor spheres formed by C4-2B cells were more in number and larger than those of LNCaP cells (Figure 6C), which is consistent with the results of a previous report [31]. The number of spheres formed by derived cells transfected with shSETD1A was significantly reduced compared to that of the control C4-2B cells, and representative images of the spheres showed that the size of the spheres derived from shSETD1A was smaller than that of the control (Figure 6D and Figure S11). Changes in *OCT4* gene expression were particularly significant in cancer stem cells. C4-2B cells expressed 60.3% more *OCT4* compared to LNCaP cells. Cancer stem cells (CSCs) derived from C4-2B cells showed 2.1 times more *OCT4* gene expression than C4-2B cells (Figure 6E). *OCT4* expression in CSCs derived from C4-2B cells was significantly inhibited upon SETD1A depletion (Figure 6F), indicating that SETD1A may play a critical role in the proliferation of cancer stem cells of mCRPC. 

Next, we sought to determine the clinical relevance of the relation between SETD1A–FOXM1 expression and prognosis in prostate cancer. SurvExpress (Interface v2.0), an online tool, was used to determine the risk assessment in cancer datasets to analyze the prognostic role of SETD1A–FOXM1 in different risk groups. In the dataset analyzed [34], 140 patients with prostate cancer, including mCRPC, were divided into high-risk (*n* = 70) and low-risk (*n* = 70) groups according to the prognostic index. The high-risk group patients displayed a significantly higher level of both *SETD1A* and *FOXM1* expression, compared to the low-risk group of patients (Figure 6G). Furthermore, patients with high expression of both the genes showed a significantly (*p* = 0.0017) poor RFS outcome compared to those with low expression of both the genes (Figure 6H), indicating that SETD1A–FOXM1 act as potential biomarkers for predicting the prognosis of patients with mCRPC.

## 3. Discussion

Recently, many reports have suggested cellular and molecular mechanisms involved in the development of mCRPC. These include AR-dependent pathways (e.g., expression of AR amplification, mutations, and AR splicing variants) and AR-independent pathways (e.g., PI3K–Akt–mTOR and SRC pathways) [3]. Here, we report that the epigenetic factor SETD1A promotes the proliferation of mCRPC through FOXM1. First, it was confirmed that SETD1A, which is involved in the growth of various prostate cancer cells, was overexpressed in mCRPC and activates FOXM1 signaling during the progression of mCRPC. Activation of FOXM1 by SETD1A in mCRPC is due to direct regulation of the transcription of *FOXM1* by SETD1A. SETD1A is recruited to the *FOXM1* promoter upon binding to E2F1, where it induces H3K4me3 and modulates the transcription of the *FOXM1* gene by modifying the chromatin structure. Several studies have reported on the role of FOXM1 in the proliferation of cancer cells and the development of anticancer drug resistance [35,36]. In addition, FOXM1 has been predicted by computational modeling analysis as being one of the transcription factors that are active in mCRPC [9]. However, the detailed mechanism by which the upregulation of FOXM1 is regulated in mCRPC is unclear. Thus, the regulation of *FOXM1* expression by SETD1A suggests that SETD1A may play a key role in prostate cancer growth, metastasis, and castration resistance. Indeed, this study showed that SETD1A plays an important role in the proliferation, migration, invasion of C4-2B cells, and cancer stem cell formation. We used LNCaP cells and LNCaP-derived metastatic castration-resistant cells (C4-2B), which have been suggested as an excellent model to study the development of mCRPC [37]. To overcome the limitation of using a single type of cell line and its castration-resistant derivatives, some key experiments were verified using various types of CRPC, and public datasets and clinical sample data were examined to prove the correlation in patients. Overexpression of SETD1A was significantly correlated with overexpression of FOXM1 (GSE50936, GSE70769) [38,39] and low survival rates of prostate patients (GSE40272) [40]. In addition, both *SETD1A* and *FOXM1* were found to be overexpressed in mCRPC compared to their expression in primary prostate tumors (GSE35988, GSE32269) [41,42].

Recently, WDR5, a subunit of the SET1/MLL (mixed lineage leukemia) complex, has been suggested as a major driver of AR-dependent prostate cancer [43] and has been shown to interact with H3T11P to guide MLL1 to the AR target gene site and promote H3K4me3. In subsequent studies, the MLL complex was also identified as a potential target for the treatment of mCRPC in AR-dependent patients [44]. Presently, we demonstrated the mechanism by which SETD1A regulates the FOXM1 pathway and showed that it does not affect the AR pathway in prostate cancer cells. Similarly, we have previously reported that SETD1A is involved in resistance to selective estrogen receptor modulators (SERMs), such as tamoxifen. In ER-positive breast cancer, SETD1A bypasses ER target gene expression, leading to gene expression involved in cell growth and metastasis [21]. The MLL complex and its association with WDR5 shows that SETD1A also plays an important role in prostate cancer, especially in the progression of mCRPC.

FOXM1 is a member of the Forkhead box (Fox) protein family characterized by a common DNA-binding domain (DBD) termed the winged-helix domain [28]. FOXM1 functions as a transcription factor and its upregulation has been reported in numerous cancers [45]. In prostate cancer, FOXM1 is required for the pathogenesis of prostate tumors, and its expression is regulated by *MYC* [46]. Consistently, another report suggested that FOXM1 is essential for carcinogenesis as it controls the expression of 11β-Hsd2, which is important for tumor cell proliferation [47]. Furthermore, FOXM1 has been observed to drive prostate cancer metastasis or malignancy by forming the COUP–TFII–FOXM1–CENPF cascade [48] or by cooperating with CENPF [49]. Importantly, high FOXM1 expression level has been correlated with advanced tumor stages, high Gleason score, and poor prognosis [50]. Accordingly, targeting the FOXM1 pathway using the novel inhibitor Monensin is considered as an efficient therapeutic strategy for mCRPC [33].

The transcription of FOXM1 is regulated by several identified transcription factors. E2F1 is a well-characterized transcription factor that controls FOXM1 expression, and even in this study, we confirmed that E2F1 binds to the *FOXM1* promoter region and directly regulates FOXM1 expression in C4-2B cells. Previous studies using gel filtration chromatography showed only a potential association of SETD1A with E2F1 [51]. However, our study clearly showed that SETD1A binds to E2F1. In addition, SETD1A recruited to the *FOXM1* promoter region with E2F1 and E2F1 knockdown results in reduced SETD1A recruitment, suggesting that binding of E2F1 and SETD1A is a major mechanism for recruitment of SETD1A to the *FOXM1* promoter.

FOXM1 is a transcription factor that controls cell proliferation by regulating the transcription of genes involved in regulating the G1/S and G2/M phases of the cell cycle [52]. Consistently, we observed that SETD1A silencing resulted in inhibition of the cell cycle in prostate cancer cell lines. Most importantly, FOXM1, at high expression levels, is known to act as a driver of prostate cancer malignancies by synergistically interacting with CENPF [49]. Thus, regulation of the FOXM1 pathway by SETD1A may be sufficient to control mCRPC development. Together with previous studies showing that targeting the FOXM1 pathway may be a new therapeutic strategy for treating enzalutamide-resistant CRPC [33], our findings have raised interesting possibilities that indicate that SETD1A may be a new target for the development of therapeutics for mCRPC. Although FOXM1 signaling was amplified in mCRPC, FOXM1 signaling also appeared to play an important role in LNCaP cells, an AR-dependent prostate cancer cell line. Thus, targeting *FOXM1* expression by inhibiting SETD1A may serve as a fundamental alternative to treating prostate cancer.

## 4. Materials and Methods 

### 4.1. Cell Culture and Western Blotting

LNCaP, PC-3, DU145, and LNCaP-LN3 cell lines were purchased from the Korean Cell Line Bank (KCLB, Seoul, Korea). The C4-2B cell line was kindly provided by Professor Jaehong Kim (Gachon University, Incheon, Korea). LNCaP, PC-3, DU145, and C4-2B cell lines were cultured in Roswell Park Memorial Institute (RPMI) 1640 medium (HyClone, South Logan, UT, USA) supplemented with 2.05 mM L-glutamine and 10% fetal bovine serum at 37 °C and 5% CO_2_. The LNCaP-LN3 cell line was maintained in Minimum Essential Medium (Gibco, Grand Island, NY, USA) containing Earle’s salts, L-glutamine, and 10% fetal bovine serum at 37 °C and 5% CO_2_. Western blotting was performed as described in a previous study [53]. Antibodies used for the Western blotting are listed in Table S1. Original blots with densitometry data can be found at Figure S12.

### 4.2. Data Collection and Analysis of the Transcriptome Datasets

SETD1A mRNA expression in prostate tissues was determined through the analysis of the Tomlins dataset, which is available at Oncomine (http://www.oncomine.org). The PROGgene platform (http://genomics.jefferson.edu/proggene/) was used to estimate the prognostic value of SETD1A expression in prostate cancer. The data used for the comparison of SETD1A and FOXM1 mRNA levels in primary and CRPC were obtained from GEO (Gene Expression Omnibus) (https://www.ncbi.nlm.nih.gov/geo/). The R2 Genomics Analysis and Visualization platform (http://r2.amc.nl) was used to investigate the correlation between SETD1A and FOXM1 mRNA expression in prostate cancer microarray datasets (GSE50936). SurvExpress (http://bioinformatica.mty.itesm.mx/SurvExpress) was used to determine the prognostic value of SETD1A and FOXM1 mRNA expression and provide their risk assessment in prostate cancer. A Kaplan–Meier survival analysis was performed for the Taylor MSKCC (Memorial Sloan Kettering Cancer Center) Prostate dataset (GSE21032).

### 4.3. Cell Proliferation Assay

Cell viability was determined according to a previously published method [21]. Briefly, cells were seeded in a 6-well (7 × 10^4^/well) plate and incubated at 5% CO_2_ and 37 °C. After the cells were transfected with non-specific siRNA (siNS) or siRNA targeting SETD1A (siSETD1A), the plates were transferred to the IncuCyte^®^ ZOOM system (Essen Bioscience, Ann Arbor, MI, USA), which allows automated in-incubator monitoring of live cells. Images were captured at 2-h intervals using the algorithm of the IncuCyte ZOOM software (Ver. 2013B). The final cell growth curves were generated using the mean values of cell density measured at 16 sites in each well for 3 or 5 days. For colony-formation assays, shNS- or shSETD1A-expressing cells were resuspended in RPMI-1640 medium containing 0.35% agarose and plated onto a 0.5% agarose underlay in 6-well culture plates at a density of 5 × 10^3^ cells per well. After incubating for 2 weeks at 37 °C in a 5% CO_2_ incubator, the colonies were stained using crystal violet (Sigma–Aldrich, St. Louis, MO, USA), washed with PBS (Phosphate-Buffered Saline), and imaged. The number of visible colonies was counted. 

### 4.4. RNA Interference and Quantitative Reverse Transcription PCR

Transfection was performed using Oligofectamine (Invitrogen, Carlsbad, USA) according to the manufacturer’s protocol. The sequences of siRNAs are listed in Table S2. LNCaP and C4-2B cells expressing control shRNA (pLKO.1-puro) or shRNA-targeting SETD1A (Sigma: TRCN0000156170) were generated via lentiviral transfection and puromycin (Sigma–Aldrich, St. Louiscity, MO, USA) (2 µg/mL) selection. SMARTVector inducible mCMV-TurboGFP human SETD1A shRNA targeting the 3′UTR of SETD1A (Dharmacon, Lafayette, CO, USA) was used to generate stable cell lines expressing shRNA against SETD1A in a doxycycline (Sigma-Aldrich, St. Louis, MO, USA)-inducible manner via lentiviral transfection and puromycin (2 µg/mL) selection. shRNAs were induced with Dox (1 µg/mL) for the following indicated experiments. For RT-qPCR, total RNA was isolated using Trizol (Invitrogen, Carlsbad, CA, USA). RNA was subjected to reverse transcription using the iScript cDNA synthesis kit (Bio-Rad Laboratories, Hercules, CA, USA) in a total reaction volume of 20 μL. 2 μL of the product (cDNA) was used for qPCR, which was performed on a LightCycler 480 II (Roche, Indianapolis, IN, USA) using the primers listed in Table S3. Results shown are the mean and range of variation of PCR reactions performed in triplicate using the same cDNA sample. Expression levels were normalized to the expression levels of *18S* rRNA.

### 4.5. RNA Sequencing and Gene Expression Analysis

LNCaP and C4-2B cells were transfected with shRNA against SETD1A, and RNAs were extracted using the RNeasy Mini Kit (Qiagen, Hilden, Germany), according to the manufacturer’s instructions. RNA-seq libraries were prepared using a TruSeq Stranded mRNA kit (Illumina, San Diego, CA, USA). Each of the libraries prepared was sequenced using an Illumina NextSeq 500 system (Illumina, San Diego, CA, USA). The original image data was converted into sequence data and stored in the FASTQ format. Genes with absolute fold changes of at least 1.5 and *p* < 0.05 between groups were considered to be differentially expressed. Gene set enrichment and pathway analysis were carried out using Enrichr [54]. Gene set enrichment analysis (GSEA) [55] was used to analyze the enrichment of FOXM1-dependent genes collected from previous reports [22,23].

### 4.6. Chromatin Immunoprecipitation (ChIP)

LNCaP and C4-2B cells were transfected with siRNAs and incubated for 72 hours at 5% CO_2_ and 37 °C. At approximately 90% confluency, the cells were crosslinked with 1% formaldehyde, followed by sonication to shear chromatin fragments. Immunoprecipitation of the sonicated chromatin was performed by incubating it with an antibody overnight at 4 °C. Antibodies used for the ChIP assay are listed in Table S1. Crosslinking was reversed by heating the solution overnight at 65 °C, and protein-associated DNA sequences were purified by phenol–chloroform extraction and ethanol precipitation. The purified DNA sequences were dissolved in nuclease-free water and analyzed by real-time quantitative PCR using the LightCycler 480 II system and SYBR Green I Master (Roche, Indianapolis, IN, USA). Results shown are the mean and range of variation of PCR reactions of a single experiment performed in triplicate. Importantly, results were expressed as a percentage of input chromatin (before immunoprecipitation). The primers used for amplifying the FOXM1 promoter are shown in Table S4.

### 4.7. Formaldehyde-Assisted Isolation of Regulatory Elements (FAIRE)-qPCR

FAIRE-qPCR was performed as previously described [56]. Briefly, LNCaP and C4-2B cells were transfected with siRNAs and cultured for 3 days. At ~90% confluency, the cells were crosslinked with 1% formaldehyde, and cell extracts were prepared. Results shown are the mean and range of variation of PCR reactions performed in triplicate using the same DNA sample. Importantly, results are expressed as a percentage of input chromatin (Input DNA). Sequences of FAIRE-qPCR primers are the same as those used for ChIP-qPCR analysis. 

### 4.8. Coimmunoprecipitation

HA-fused E2F1 (pCMV-HA-E2F1, 24225) was purchased from Addgene (Watertown, MA, USA). pcDNA3-flag-SETD1A was kindly provided by Professor David Skalnik (Purdue University). Plasmids (total 8 μg) were transfected into LNCaP or C4-2B cells using Lipofectamine 2000 (Invitrogen, Carlsbad, CA, USA) according to the manufacturer’s instructions. Cell extracts were prepared in RIPA (Radioimmunoprecipitation assay) buffer (50 mM Tris-Cl (pH 8.0), 150 mM NaCl, 1% NP-40, 1% sodium deoxycholate, 0.1% sodium dodecyl sulfate, 2 mM EDTA (Ethylenediaminetetraacetic acid)) after 72 h of transfection. The coimmunoprecipitation (CoIP) experiments and immunoblotting were performed as described previously [57]. Antibodies used for the CoIP assays are listed in Table S1. 

### 4.9. Cell Cycle Assay

After transfection with siRNA for the indicated time, cells were suspended in PBS in a 15 mL conical tube (SPL Life Sciences, Gyeonggi-do, Korea). Then, the cells were fixed using 5 mL cold 70% EtOH at 4 °C for 1 h. The fixed cells were washed and resuspended in 0.5 mL PBS and incubated with propidium iodide (PI; 50 μg/mL) and RNase A (0.1 mg/mL) at 37 °C for 30 min. The cells were then analyzed using FACSCalibur (BD Biosciences, San Jose, CA, USA). 

### 4.10. Cell Migration and Invasion Assay

Migration assays were performed using a 24-well Transwell chamber (Costar 3422, Corning Inc, Corning, NY, USA). Invasion assays were conducted using BioCoat™ Matrigel® Invasion Chambers (354480, Corning Inc., Corning, NY, USA). Briefly, after transfection, 100 μL of serum-free RPMI 1640 medium and 200 μL of LNCaP or C4-2B cells (1.5 × 10^5^ cells/mL) suspended in serum-free RPMI 1640 medium were added to the upper chambers. The cells were allowed to migrate for 24 h or invade for 48 h toward the lower chamber that contained 750 µL of medium supplemented with 10% FBS (fetal bovine serum) (used as a chemoattractant). Then, the cells were fixed with methanol for 20 min, stained with 0.1% crystal violet for 15 min, and washed with PBS. The cells remaining on the upper membrane were removed with cotton wool, whereas the cells that had migrated or invaded were imaged with a Nikon TS100 stereomicroscope coupled to a Canon G10/G11 camera (Canon, Tokyo, Japan). Three random field images per insert were captured for quantification. Separately, 10% acetic acid was used to dissolve the stain and absorbance of the cells was then measured at 570 nm using Synergy H1 Hybrid Reader (BioTek, Winooski, VT, USA).

### 4.11. Tumor Sphere Formation Assay

Cells (200 cells/well) were seeded in each well of a 96-well ultra-low attachment plate (Corning Inc, Corning, NY, USA). The cells were cultured in serum-free RPMI-1640 medium supplemented with 20 ng/mL epidermal growth factor (EGF; Sigma-Aldrich, St. Louis, MO, USA), 10 ng/mL basic fibroblast growth factor (FGF; Sigma-Aldrich, St. Louis, MO, USA), 5 μg/mL insulin (Invitrogen, Carlsbad, CA, USA), 0.4% bovine serum albumin (VWR Life Science, Radnor, PA, USA), and B-27^®^ Supplement (Invitrogen, Carlsbad, CA, USA). The upper and lower edges of the 96-well plate were sealed with a tape to avoid evaporation of the medium. The cells were incubated at 37 °C and supplied with 5% CO_2_ for 1 week. After the formation of tumor spheres, the number of the spheres was counted under a microscope. 

### 4.12. Statistical Analysis

The results of RT-qPCR, ChIP-qPCR, FAIRE-qPCR, cell proliferation, cell cycle, migration, invasion, soft agar colony formation, and sphere formation experiments were statistically analyzed by the Student’s two-tailed *t*-test (* *p* < 0.05).

## 5. Conclusions

Our study illustrates that SETD1A plays a critical role in CRPC. During the castration resistance process, the expression level of SETD1A is elevated, and overexpressed SETD1A enhances *FOXM1* transcription via binding to E2F1. The SETD1A depletion impaired cell proliferation, cell migration, invasion, and sphere formation in CRPC cells. Furthermore, the SETD1A–FOXM1 axis is related to prognosis in prostate cancer clinically, suggesting a potential therapeutic target for mCRPC.

## Supplementary Materials

The following are available online at https://www.mdpi.com/2072-6694/12/7/1736/s1, Figure S1: SETD1A depletion inhibits prostate cancer cells growth, Figure S2: SETD1A-dependent genes are enriched in cell cycle pathway, Figure S3: Increase in SETD1A effect on FOXM1 target gene expression in C4-2B cells compared to that in LNCaP cells, Figure S4: The effect of SETD1A depletion on the expression of AR target genes in LNCaP cells, Figure S5: The effect of SETD1A depletion on the expression of AR target genes in C4-2B cells, Figure S6: Correlation analysis of SETD1A and FOXM1 expression, Figure S7: Regulation of FOXM1 expression by E2F1 in C4-2B cells, Figure S8: Overexpression of SETD1A and FOXM1 in primary and CRPC tissue samples, Figure S9: SETD1A depletion affects cell cycle, proliferation, and migration in LNCaP cells, Figure S10: Inhibition of C4-2B cells invasion by SETD1A depletion, Figure S11: Inhibition of sphere formation in C4-2B by SETD1A depletion, Figure S12: Original blots with densitometry data, Table S1: Antibodies used for Western blotting, coimmunoprecipitation, and ChIP assay, Table S2: Sequences of siRNAs and shRNAs, Table S3: Primer sequences for RT-qPCR, Table S4: Primer sequences for FAIRE-qPCR and ChIP.
