# Supplementary material for: SETD1A Promotes Proliferation of Castration-Resistant Prostate Cancer Cells via FOXM1 Transcription
Source: Cancers (Basel). 2020 Jun 30;12(7):1736. doi: 10.3390/cancers12071736 (PMC7407996; doi:10.3390/cancers12071736)
Supplement: Supplementary file 1 [file cancers-12-01736-s001.pdf]

Supplementary Materials: SETD1A Promotes Proliferation of Castration-Resistant Prostate Cancer Cells via FOXM1 Transcription

Liu Yang, Mingli Jin, Sung Jean Park, Seung-Yong Seo and Kwang Won Jeong

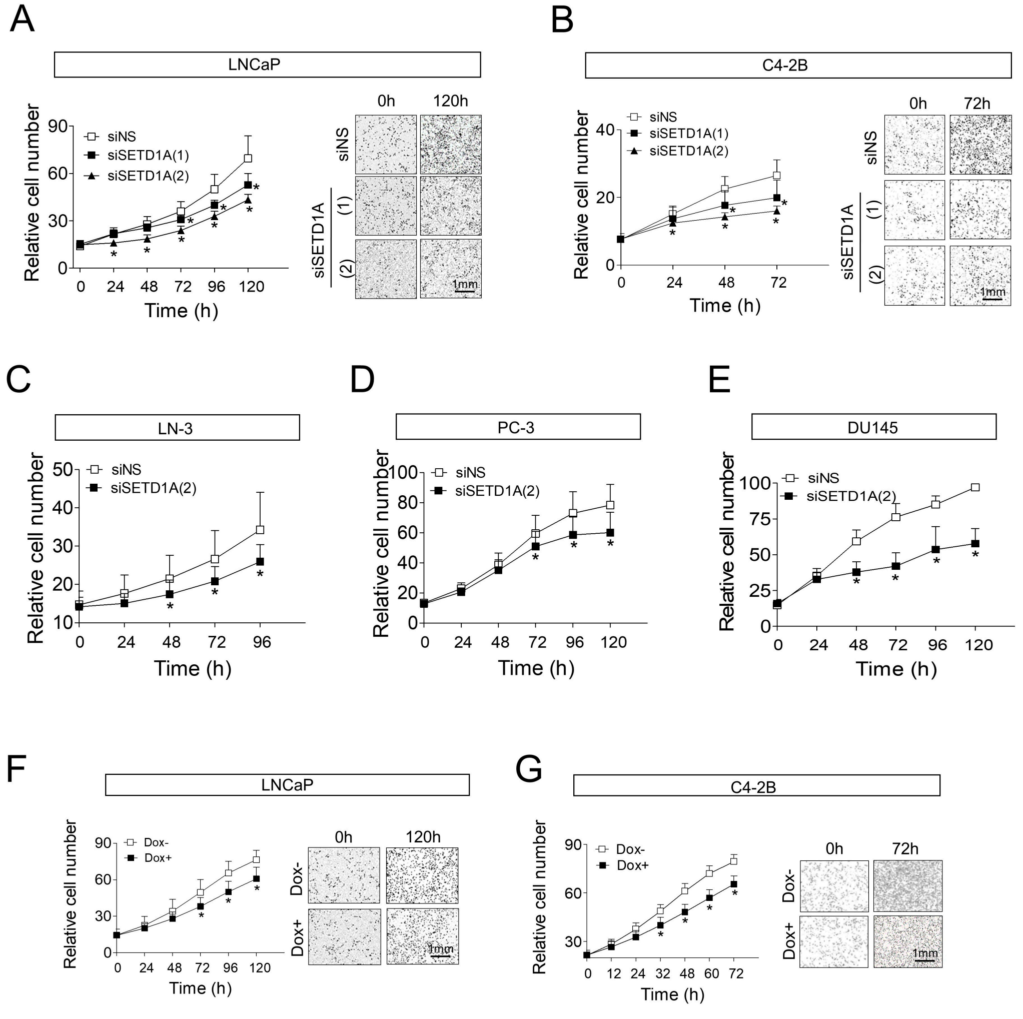

Figure S1. SETD1A depletion inhibits prostate cancer cells growth. (**A**,**B**) Effect of SETD1A depletion on the proliferation of LNCaP (**A**) and C4-2B cells (**B**) using siRNAs targeting different site of SETD1A mRNA (siSETD1A(1) and siSETD1A(2)). (**C**–**E**) Proliferation of other types of prostate cancer cell lines LN-3 (**C**), PC-3 (**D**) and DU145 (**E**) cells transfected with siNS or siSETD1A(2) were monitored by IncuCyte. Data represents the mean ± S.D. *$p < 0.05$. (**F**,**G**) The SETD1A depletion by Tet-inducible knockdown system on cell growth. Depletion of SETD1A was induced by treatment of doxycycline (1μg/mL) during experiments period in LNCaP or C4-2B cells. Cell growth was monitored by live-imaging system for 72 or 120 hours. Data are mean ± S.D. *$p < 0.05$.

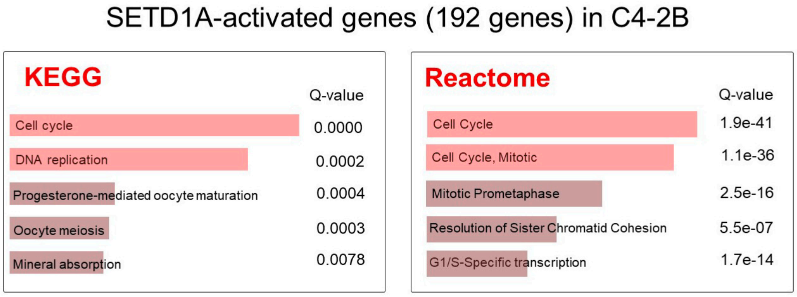

Figure S2. SETD1A-dependent genes are enriched in cell cycle pathway. Pathway enrichment was analyzed using SETD1A activated genes in C4-2B cells. The length of the bars represent the combined score computed by taking the log of the *p*-value form Fisher exact test and multiplying that by the z-score of deviation from the expected rank.

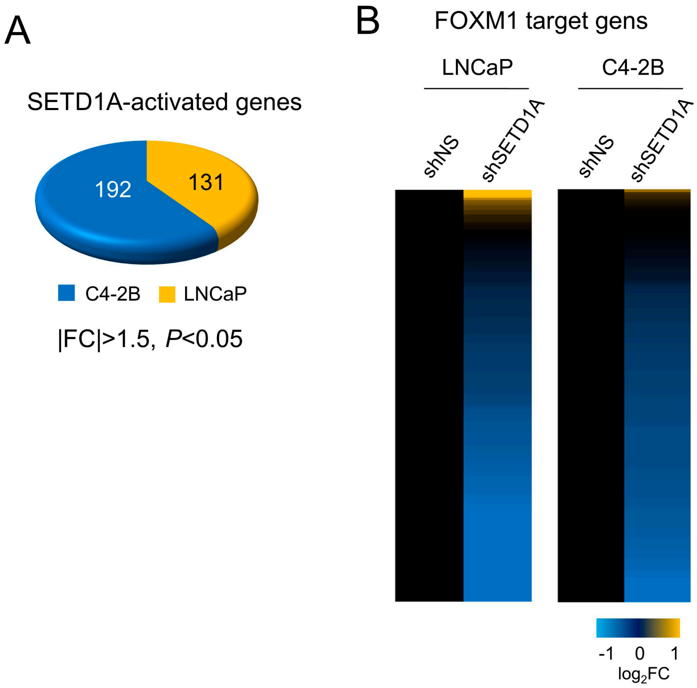

Figure S3. Increase of SETD1A effect on FOXM1 target gene expression in C4-2B cells compared to that in LNCaP cells. (**A**) The numbers of SETD1A activated genes in LNCaP (yellow pie) and C4-2B (blue pie) cells. (**B**) The effect of SETD1A depletion on the expression of FOXM1 target genes in LNCaP and C4-2B cells.

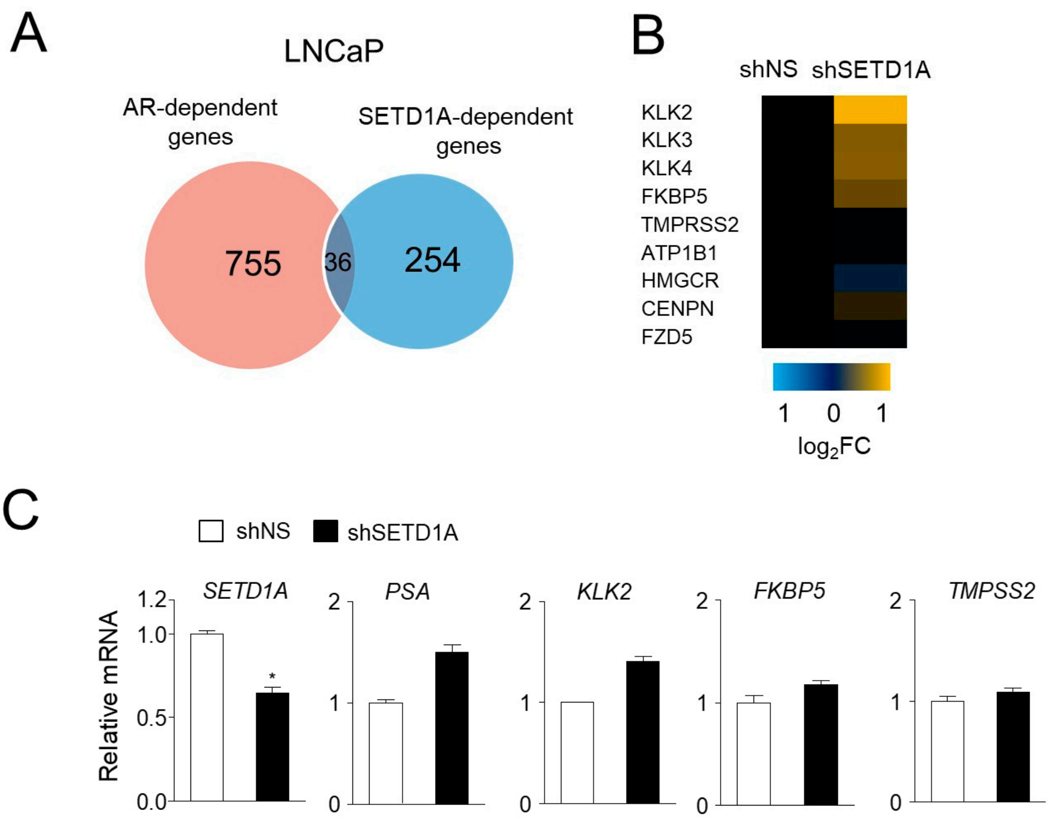

Figure S4. The effect of SETD1A depletion on the expression of AR target genes in LNCaP cells. (**A**) Venn diagram showing overlap of AR target genes and SETD1A-dependent genes in LNCaP cells. (**B**) Heat map for representative AR target genes expression from RNA-seq in LNCaP cells. (**C**) Validation of the effect of SETD1A depletion on mRNA levels of AR target genes in LNCaP cells using RT-qPCR. Data are mean ± S.D, $^*p < 0.05$.

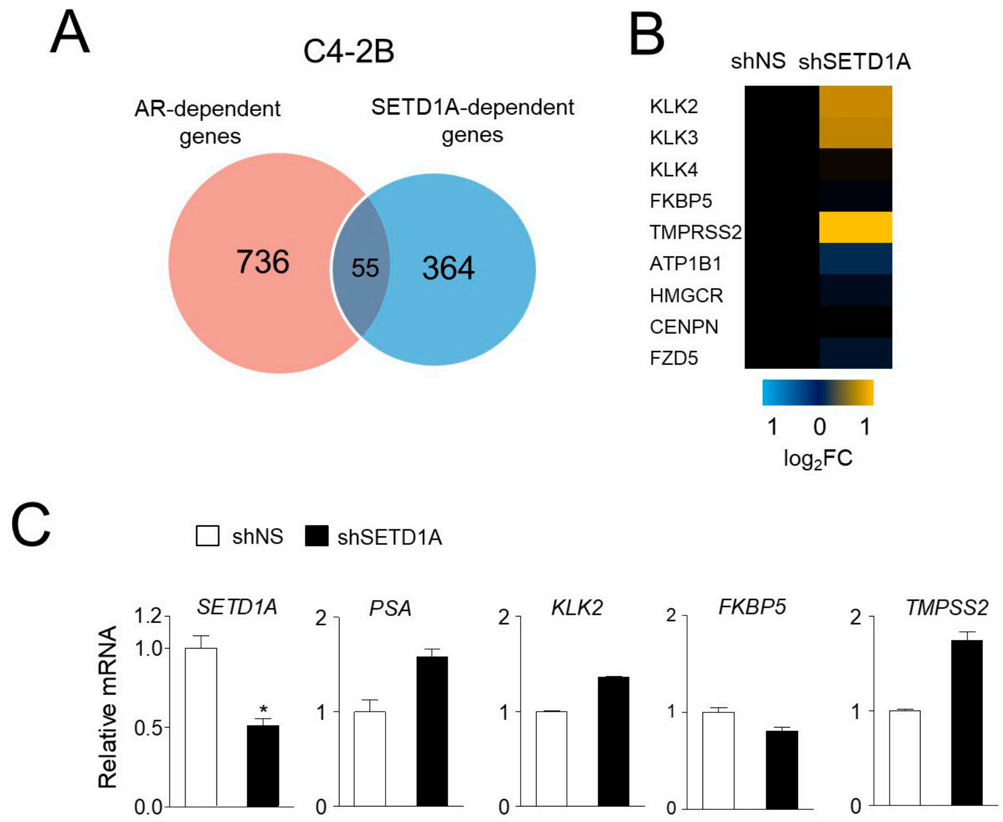

Figure S5. The effect of SETD1A depletion on the expression of AR target genes in C4-2B cells. (**A**) Venn diagram showing overlap of AR target genes and SETD1A-dependent genes in C4-2B cells. (**B**) Heat map for representative AR target genes expression from RNA-seq in C4-2B cells. (**C**) Validation of the effect of SETD1A depletion on mRNA levels of AR target genes in C4-2B cells using RT-qPCR. Data are mean ± S.D, $^{*}p < 0.05$.

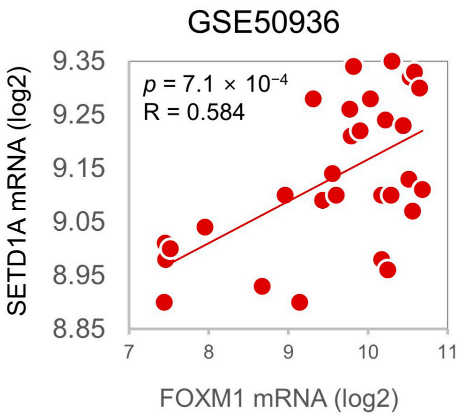

Figure S6. Correlation analysis of SETD1A and FOXM1 expression. Pearson correlation analysis of SETD1A and FOXM1 expression in LNCaP (androgen dependent), VCaP (androgen dependent and osteoblastic bone metastases), and DuCaP (androgen dependent and osteoblastic bone metastases) was conducted through R2 platform. Plotted data showing the level of log_2 mRNA expression in GSE50936 dataset.

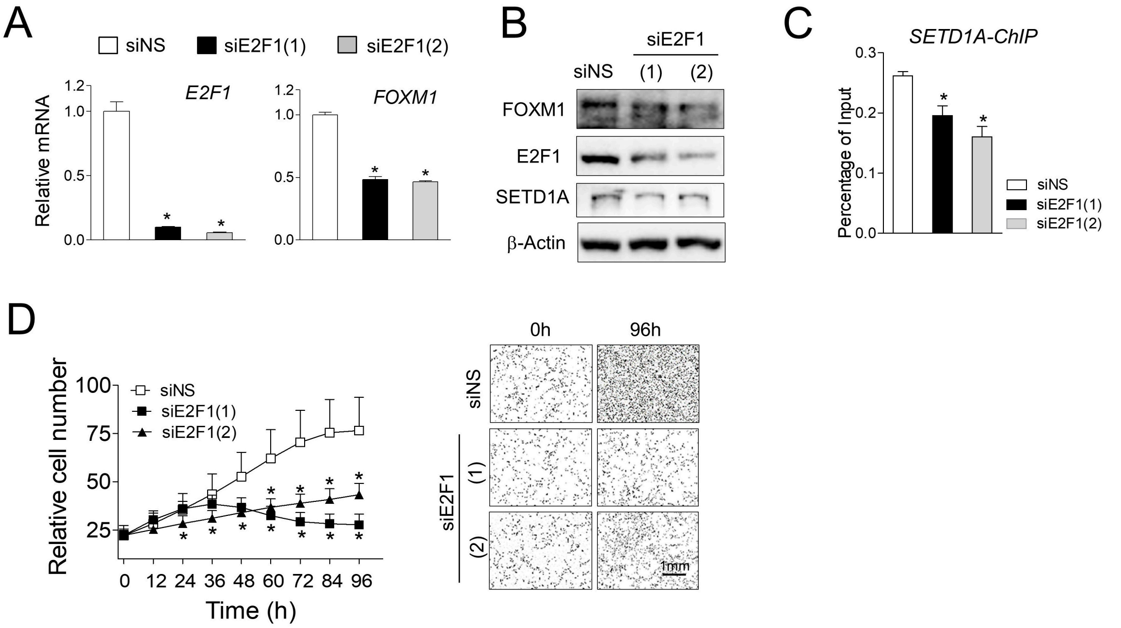

Figure S7. Regulation of FOXM1 expression by E2F1 in C4-2B cells. (**A**,**B**) C4-2B cells were transfected with siNS or siRNA targeting two different site of E2F1 mRNA (siE2F1 (1) and siE2F1 (2)). Three days after transfection, mRNA and protein level of E2F1 was analyzed by RT-qPCR (**A**) and western blot (**B**). (**C**) SETD1A recruitment was analyzed by ChIP-qPCR in E2F1-depleted C4-2B cells. Data was normalized by input. (**D**) The effect of E2F1 depletion on cell growth of C4-2B. Data represent mean ± S.D. *$p < 0.05$.

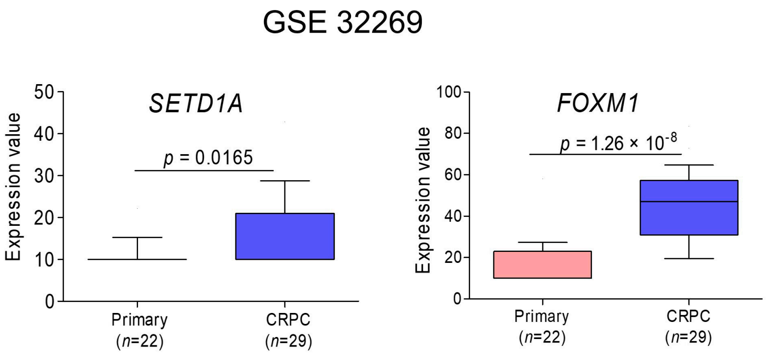

Figure S8. Overexpression of SETD1A and FOXM1 in primary and CRPC tissue samples. SETD1A and FOXM1 expression level in primary and castration resistant prostate cancer tumor tissues were collected from GSE32269 dataset.

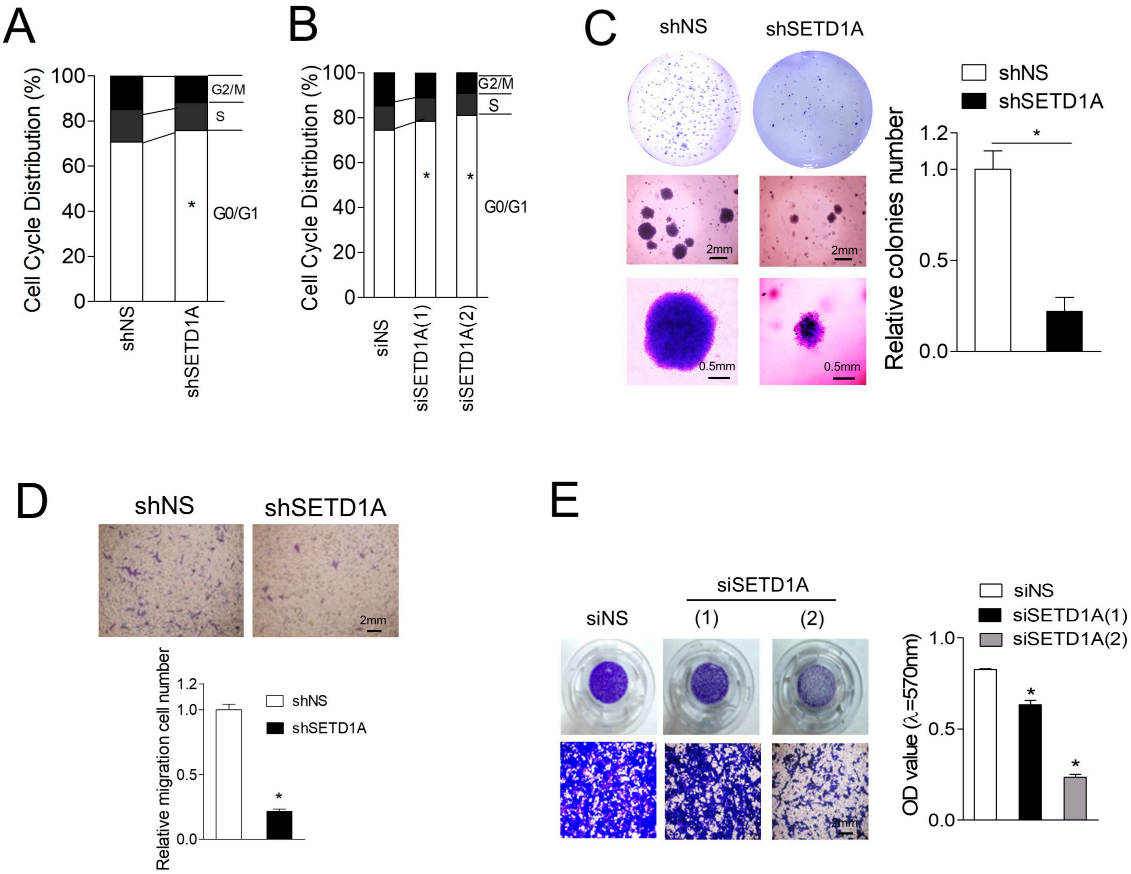

Figure S9. SETD1A depletion affects cell cycle, proliferation, and migration in LNCaP cells. (**A,B**) Cell cycle distribution of LNCaP cells after depletion of SETD1A by shRNA (**A**) or siRNAs (**B**). (**C**) LNCaP cells transfected with shNS or shSETD1A, colony formation assay was performed in soft agar. Images were taken under microscope (left panel) and colony numbers in shNS or shSETD1A transfected cells were presented in bar graph. (**D,E**) Migration assay of SETD1A-depleted LNCaP cells by shRNA (**D**) or siRNAs (**E**). The migrated cells were stained by crystal violet and imaged and counted under a microscope (bottom panel). Bar graph represents the quantitative analysis of stained migrated cells using a microplate reader at 570 nm (right panel). Data represent mean ± S.D. *$p < 0.05$.

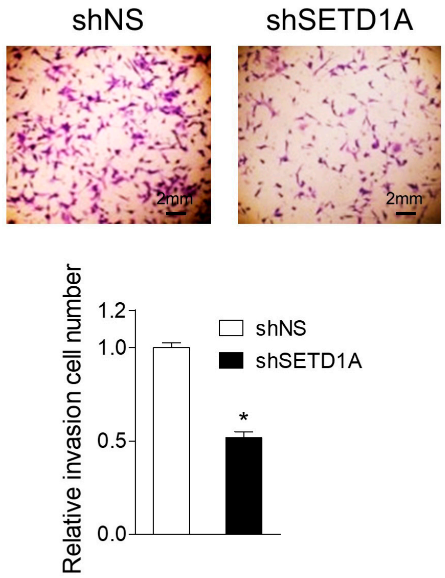

Figure S10. Inhibition of C4-2B cells invasion by SETD1A depletion. Cell invasion assay were performed in shNS or shSETD1A transfected C4-2B cells using matrigel transwell system. The invaded cells were monitored by microscope and quantified by staining with crystal violet. Date represent mean ± S.D. $*p < 0.05$.

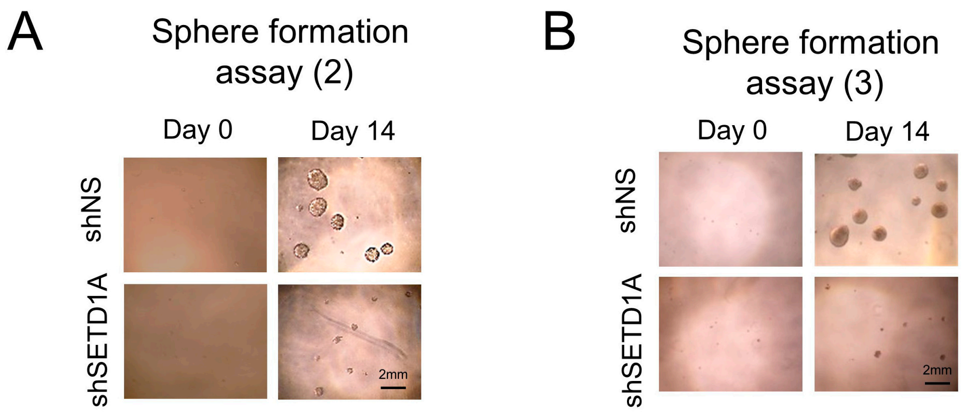

Figure S11. Inhibition of sphere formation in C4-2B by SETD1A depletion. **(A,B)** Repeat of sphere formation assay performed in Figure 6D using C4-2B cells transfected with shNS or shSETD1A. Representative images showing the difference of formed spheres in indicated conditions.

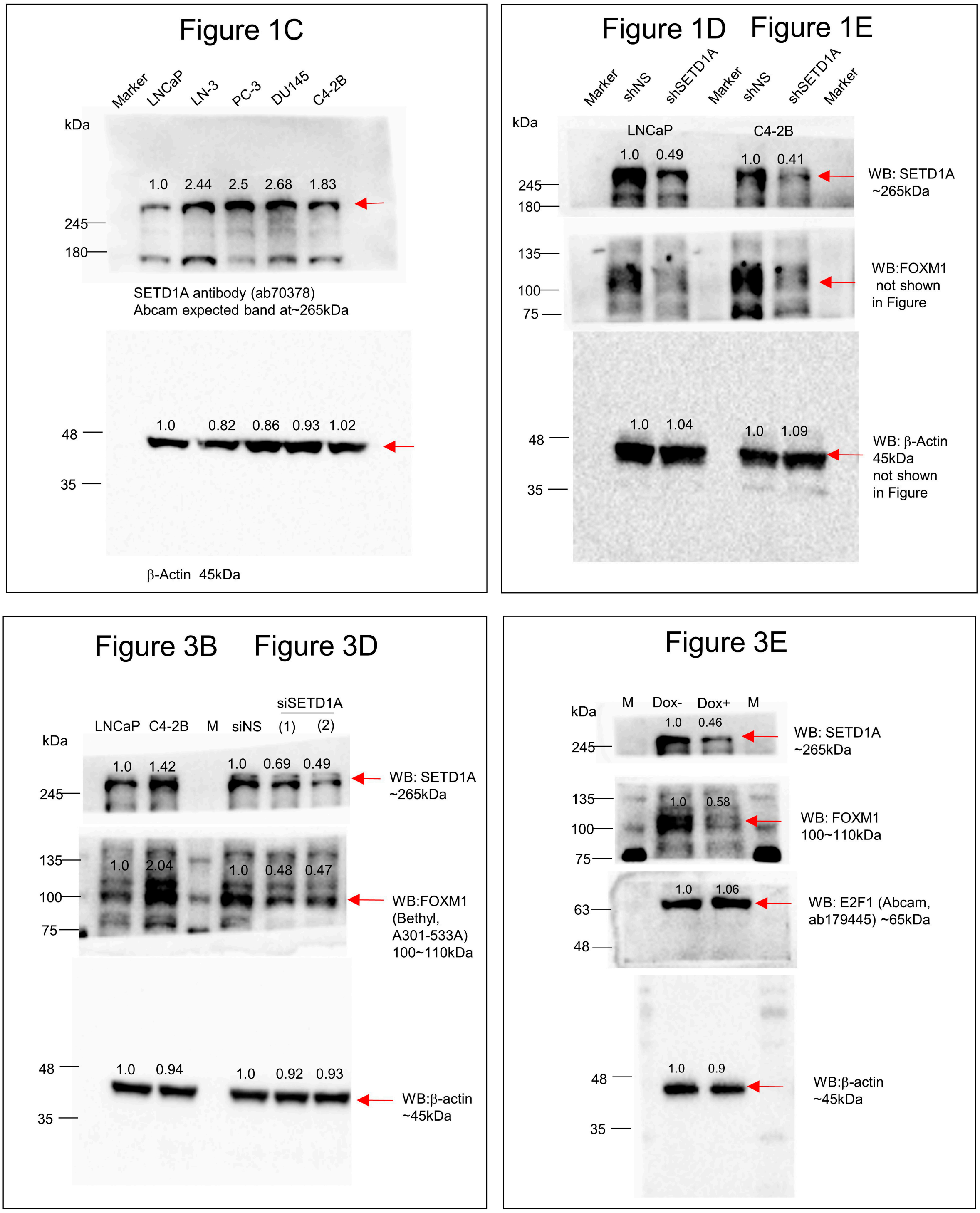

Figure S12. Original blots with densitometry data.

Figure S12 . *Cont.*

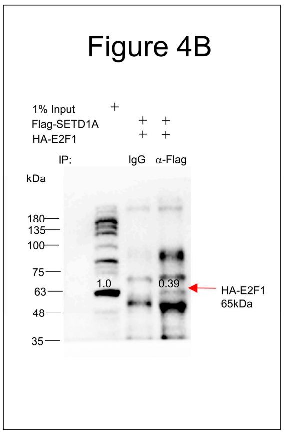

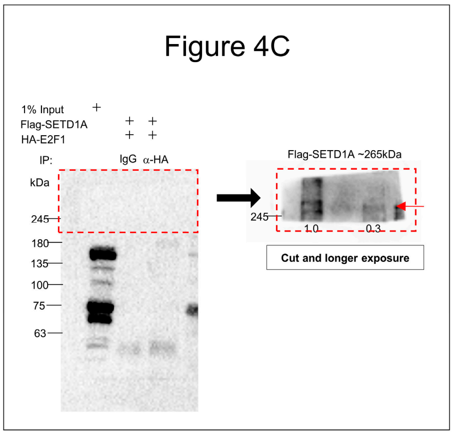

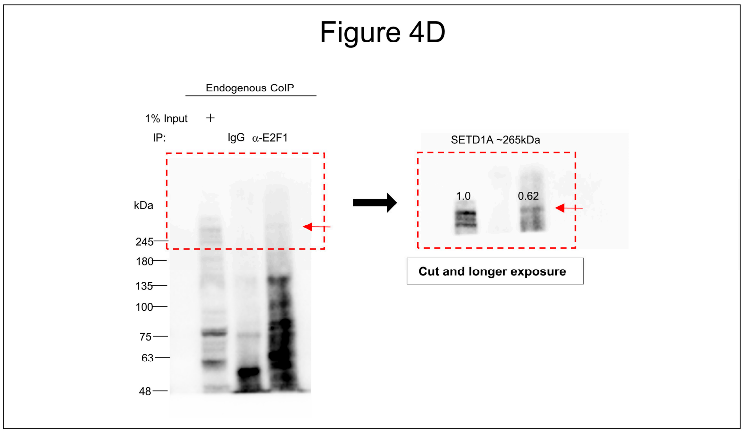

Figure S12. *Cont.*

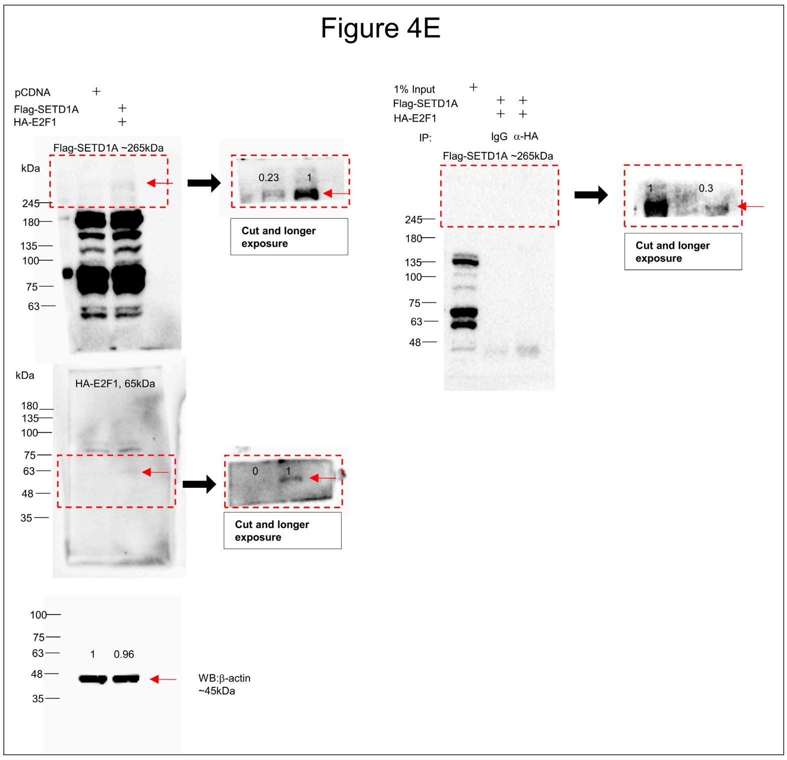

Table S1. Antibodies used for western blotting, coimmunoprecipitation, and ChIP assay.

Antibody	Vendor	Catalog number	Use
β-actin	Thermo Fisher Scientific	MA1-140	WB
SETD1A	Abcam	ab70378	WB
FOXM1	Bethyl Laboratories	A301-533A	WB
E2F1	Abcam	ab179445	WB, CoIP
Mouse IgG	Santa Cruz Biotechnology	sc-2025	CoIP, ChIP
Rabbit IgG	Cell Signaling Technology	2729	CoIP, ChIP
Histone H3K4me3	Active Motif	39159	ChIP
Pol II	Merck Millipore	05-623	ChIP
E2F1	Santa Cruz Biotechnology	sc-251X	ChIP
SETD1A	Bethyl Laboratories	A300-289A	ChIP
α-HA	Santa Cruz Biotechnology	sc-7392	WB,CoIP
α-Flag	Sigma-Aldrich	F7425	WB,CoIP

Table S2. Sequences of siRNAs and shRNAs.

Name	Sequence
shNS	CCGGCAACAAGATGAAGAGCACCAACTCGAGTTGGTGCTCTTCATCTTGTTGTTTTT
shSETD1A	CCGGCGGAAGAAGAAGCTCCGATTTCTCGAGAAATCGGAGCTTCTTCTTCCGTTTTTTG
siNS	UUCUCCGAACGUGUCACGUdTdT (sense)<br>ACGUGACACGUUCGGAGAAdTdT (anti-sense)
siSETD1A(1)	CUUUGCGGAGAAGAAGCUGdTdT (sense)<br>CAGCUUCUUCUCCGCAAAGdTdT(anti-sense)

siSETD1A(2)	GCGAUUCGUCUUCCAAAUGdTdT (sense)<br>CAUUUGGAAGACGAAUCGCdTdT (anti-sense)
siE2F1(1)	CUACUCAGCCUGGAGCAAGdTdT (sense)<br>CUUGCUCCAGGCUGAGUAGdTdT (anti-sense)
siE2F1(2)	ACAUCACCAACGUCCUUGAdTdT (sense)<br>UCAAGGACGUUGGUGAUGUdTdT (anti-sense)
siFOXM1	GCCAAUCGUUCUCUGACAGdTdT (sense)<br>CUGUCAGAGAACGAUUGGCdTdT (anti-sense)

Table S3. Primer sequences for RT-qPCR.

Name	Forward	Reverse
18S	GAGGATGAGGTGGAACGTGT	TCTTCAGTCGCTCCAGGTCT
SETD1A	CAGAAGGTGTACCGCTATGATG	TCTTGGAGGTCTTCGACTGGT
FOXM1	CGTCGGCCACTGATTCTCAAA	GGCAGG GGATCTCTTAGGTTC
E2F1	AGTCCCAGCCAGTCTCTACTCA	TGCCCATCCGGGACAA
MDC1	GTAGGCCGAATGCCTGACTG	CGGAGGATAGGTGCCTTGTC
RFC3	CTTCCTTCACAACTGGCTCAT	CTTCCTTCACAACTGGCTCAT
KIFC1	GGTGCAACGACCAAAATTACC	GGGTCCTGTCTTCTTGGAAAC
FAM83D	GCCTGGCTCGTTTCCTGAA	GGAAGTGCGTCTCGACACG
GTSE1	CAGGGGACGTGAACATGGATG	ATGTCCAAAGGGTCCGAAGAA
SKA3	TGAGCGGTACATCGTATCCCA	GGGGTTACAATTACGGGCTCT
ID1	CTGCTCTACGACATGAACGG	GAAGGTCCCTGATGTAGTCGAT
PSA	TCACAGCTACCCACTGCATCA	AGGTCGTGGCTGGAGTCATC
FKBP5	AGGCTGCAAGACTGCAGATC	CTTGCCCATTGCTTTATTGG
KLK2	GCTGCCCATTGCCTAAAGAAG	TGGGAAGCTGTGGCTGACA
TMPSS2	CCTGCAAGGACATGGGCTATA	CCGGCACTTGTGTTCAGTTTC
MYC	CTCTCAACGACAGCAGCTCG	CAACATCGATTTCTTCCTCATCTTC
Nanog	AAGGTCCCGGTCAAGAAACAG	CTTCTGCGTCACACCATTGC
KLF4	CCCACATGAAGCGACTTCCC	CAGGTCCAGGAGATCGTTGAA
OCT4	GTGTTCAGCCAAAAGACCATCT	GGCCTGCATGAGGGTTTCT

Table S4. Primer sequences for FAIRE-qPCR and ChIP.

Name	Forward	Reverse
FOXM1 (prom)	CACTTCTTCCCCCACAAGC	GCGGAGCGTTAAGGTCAC
